# Hepatitis C Virus E1 and E2 Proteins Used as Separate Immunogens Induce Neutralizing Antibodies with Additive Properties

**DOI:** 10.1371/journal.pone.0151626

**Published:** 2016-03-11

**Authors:** Elodie Beaumont, Emmanuelle Roch, Lucie Chopin, Philippe Roingeard

**Affiliations:** INSERM U966, Université François Rabelais and CHRU de Tours, Tours, France; Saint Louis University, UNITED STATES

## Abstract

Various strategies involving the use of hepatitis C virus (HCV) E1 and E2 envelope glycoproteins as immunogens have been developed for prophylactic vaccination against HCV. However, the ideal mode of processing and presenting these immunogens for effective vaccination has yet to be determined. We used our recently described vaccine candidate based on full-length HCV E1 or E2 glycoproteins fused to the heterologous hepatitis B virus S envelope protein to compare the use of the E1 and E2 proteins as separate immunogens with their use as the E1E2 heterodimer, in terms of immunogenetic potential and the capacity to induce neutralizing antibodies. The specific anti-E1 and anti-E2 antibody responses induced in animals immunized with vaccine particles harboring the heterodimer were profoundly impaired with respect to those in animals immunized with particles harboring E1 and E2 separately. Moreover, the anti-E1 and anti-E2 antibodies had additive neutralizing properties that increase the cross-neutralization of heterologous strains of various HCV genotypes, highlighting the importance of including both E1 and E2 in the vaccine for an effective vaccination strategy. Our study has important implications for the optimization of HCV vaccination strategies based on HCV envelope proteins, regardless of the platform used to present these proteins to the immune system.

## Introduction

Hepatitis C virus (HCV) infection, which affects an estimated 170 million people worldwide and frequently leads to severe chronic liver disease, constitutes a major global health concern [1]. The recent introduction of direct-acting antiviral agents has considerably improved the treatment of chronic HCV infection [2], but the eradication of this viral disease is hampered by most HCV-infected subjects being unaware of their infection status and the very high cost of the new treatment, which is unaffordable in lower-income countries [3]. Moreover, it has been estimated that the world reservoir of HCV-infected individuals increases by three to four million newly infected subjects each year, and that this phenomenon is not limited to developing countries, as 18,000 new HCV infections are thought to occur annually in the USA [4]. There is therefore an urgent need for a safe, effective and affordable prophylactic vaccine that could help to control the global epidemic and decrease the burden on healthcare systems.

The immunological correlates of the successful control of HCV infection and the underlying mechanism remain unclear, but many studies in both humans and chimpanzees have demonstrated that the early establishment of vigorous, broadly cross-reactive, long-lasting CD4+ and CD8+ T-cell responses is associated with spontaneous HCV clearance [5, 6]. It is also becoming increasingly apparent that such responses alone are not sufficient, and that neutralizing antibodies targeting conformational epitopes of the E1 and E2 virion surface envelope proteins also play a major role in conferring protection against chronic HCV infection and facilitating viral clearance [7–9]. In recent years, efforts to develop prophylactic vaccination approaches have been based on attempts to enhance both the cellular and humoral arms of the adaptive immune response [10]. Various vaccine candidates have been proposed for the induction of effective cellular immunity. In particular, a vaccination strategy based on recombinant non-pathogenic live vectors expressing the HCV NS3-NS5B gene cassette and used in a multiple prime-boost regimen has been shown to be safe, well tolerated, and highly immunogenic in healthy human volunteers, with the induction of robust, cross-reactive and sustained HCV-specific CD4+ and CD8+ T cell-mediated immunity [11, 12]. As the induction of neutralizing antibody responses is the ultimate goal for the successful prevention of HCV infection [13, 14], various strategies for developing vaccine candidates have been developed, focusing on the use of the HCV E1 and E2 envelope proteins as immunogens. Promising results have been obtained in the chimpanzee model, with an adjuvanted recombinant heterodimeric E1E2 HCV envelope protein vaccine [15]. This vaccine does not generally result in sterilizing immunity after experimental challenge, but its potential efficacy for eliciting cross-neutralizing antibodies targeting epitopes highly conserved among all known major genotypes of HCV [16] and protecting against chronic HCV-associated disease has been clearly demonstrated [17]. Moreover, a phase I dose-ranging clinical trial has demonstrated that this vaccine is safe and well tolerated in healthy human volunteers [18]. However, one of the problems encountered with this vaccination approach is the anchorage of HCV envelope proteins in intracellular compartments via their transmembrane domain (TMD) [19], rendering their extraction and purification extremely difficult, and incompatible with industrial development for vaccination purposes. Strategies based on the use of truncated E1 or E2 proteins, which are then secreted, have thus been proposed [20–22]. However, such approaches have met with limited success, because the deletion of the TMD of these proteins has been shown to impair their antigenic and functional properties [23].

We recently proposed an original vaccination strategy based on HCV E1 or E2 glycoproteins fused to the heterologous hepatitis B virus (HBV) S envelope protein and self-assembling into highly immunogenic, noninfectious and secreted subviral envelope particles resembling the HBV vaccine [24, 25]. The use of HBV envelope particles as carriers of small HCV envelope protein sequences inserted into the antigenic external hydrophilic loop has already been reported [26], but the particularity of our approach is to generate vaccine particles harboring the full-length E1 and E2 proteins in an appropriate conformation for formation of the E1E2 heterodimer. These chimeric HBV-HCV subviral envelope particles presenting the genotype 1a E1 or E2 proteins have been shown to induce a strong specific antibody response to the HCV and HBV envelope proteins in immunized animals [25, 27]. The response induced by immunization with particles presenting the E1 protein was much weaker than that induced by immunization with particles presenting the E2 protein, but both types of particle induced the production of high titers of antibodies capable of neutralizing various HCV genotypes, highlighting the importance of including both E1 and E2 proteins in the vaccine for an effective vaccination strategy. However, although both the E1 and E2 proteins, exposed individually at the surface of the chimeric particles, elicited cross-neutralizing antibodies, the cross-neutralizing response induced by the E1 and E2 proteins presented together at the surface of particles in the form of an E1E2 heterodimer did not appear to be more effective, suggesting that these proteins might be less immunogenic when presented in this heterodimeric conformation.

In this study, we used our chimeric HBV-HCV envelope particle model to compare the use of E1 and E2 as separate immunogens with their use in the form of the E1E2 heterodimer, in terms of immunogenicity and the capacity to induce neutralizing antibodies. We found that anti-E1 and anti-E2 antibodies had additive neutralizing properties, and that the best strategy for inducing a dual anti-E1 and anti-E2 response is to immunize with a mixture of vaccine particles bearing E1 and E2 separately.

## Materials and Methods

### Ethical Statement

All animal immunizations were conducted by the company Agro-Bio (La Ferte Saint Aubin, France), accredited by the French Administration to conduct protocols on laboratory animals in accordance with European Directive 2010/63/EU. The protocol was approved by the Agro Bio Ethic Committee n° C2A-99 (French Ministry of Research, agreement C41-285-4).

### Immunization of New Zealand rabbits

Chimeric HBV (adw genotype)-HCV (1a genotype) envelope subviral particles bearing the entire HCV E1 and/or E2 proteins (S+E1-S, S+E2-S and S+E1-S+E2-S particles) were produced and purified, as previously described [24, 25]. Following particle purification, the efficient incorporation of the HBV-HCV chimeric E1-S and E2-S proteins in the purified particles, and the productive folding and heterodimerization of the HCV envelope proteins, were verified (S1 Fig), as described elsewhere [24, 25]. We then prepared vaccine doses, consisting of equal amounts of the S+E1-S and S+E2-S subviral particles produced separately (7.5 micrograms of each particle) or 15 micrograms of the S+E1-S, S+E2-S or S+E1-S+E2-S immunogens, quantified by a quantitative microparticle chemiluminescence HBs antigen (HBsAg) immunoassay (ARCHITECT system; Abbott Laboratories, Abbott Park, IL). Immediately before immunization, these doses were mixed with a squalene-based oil-in-water nanoemulsion (AddaVax; Cayla-InvoGen, San Diego, CA). Groups of six rabbits were immunized subcutaneously with AddaVax-adjuvanted chimeric S+E1-S+E2-S envelope particles, or with a mixture or different sequential immunization combinations of AddaVax-adjuvanted chimeric particles harboring E1 or E2 proteins separately, on days 0, 14, 28 and 42 (S2 Fig). For sequential immunization combinations, rabbits were either immunized twice with S+E1-S particles and then twice with S+E2-S particles, or vice versa. Groups of three rabbits immunized with the Addavax adjuvant alone or with Engerix, a commercially available HBV vaccine, were used as negative and positive controls, respectively. Serum samples were collected from rabbits at various time points (days 0, 14, 28, 42, and 56), to characterize the antibody responses.

### Analysis of anti-HBsAg, anti-E1 and anti-E2 antibody responses

Anti-HBsAg antibodies were quantified with the ARCHITECT system (Abbott Laboratories). Anti-E1 and anti-E2 responses were evaluated with “in-house” ELISAs, based on the use of solid phases consisting of lysates of baby hamster kidney (BHK-21) cells expressing HCV E1 or E2 proteins, which were compared with control cells expressing ß-galactosidase (ß-gal). For these experiments, the DNA sequences encoding HCV E1 or E2 envelope glycoproteins derived from a genotype 1a HCV isolate (isolate Dj) were therefore cloned into an original eukaryotic DNA expression vector (pSFV) based on the Semliki Forest Virus replicase complex, which present a high level of expression efficiency. These DNA sequences, amplified from the previously described pSFV-HCVdj plasmid (Genbank AF529293) encoding the core-E1-E2 sequence of a genotype 1a HCV [28], also contained the upstream signal sequence responsible for translocation of the E1 or E2 ectodomain into the endoplasmic reticulum lumen (amino acids 166–191 from the HCV core protein in the HCV polyprotein for E1 and amino acids 370–383 from the HCV E1 protein in the HCV polyprotein for E2) [29, 30]. Briefly, the HCVdj E1 or E2 DNA sequences (corresponding to amino acids 166–383 and 370–746 in the HCV polyprotein) were amplified by PCR, with the proofreading Phusion® High-Fidelity DNA Polymerase (New England Biolabs) and primer pairs designed to generated BamHI restriction sites at the 5’ and 3’ ends of the amplified product: E1Dj—forward primer—5’ATAGGATCCATGACAGGGAACCTTCCTGGTTG3’ / E1Dj—reverse primer -5’ATAGGATCCTTACGCATCGACGCTGGCGAAC3’ and E2Dj—forward primer -5’ATAGGATCCATGAAGGTCCTGGTGGTGCTGT3’ / E2Dj—reverse primer -5’ATAGGATCCTTACCGCCTCCGCTTGGGATAT3’. The PCR products were inserted into pGEM-T (pGEM-T Easy Vector System; Promega), excised by digestion with BamHI and inserted into the corresponding site of pSFV (Invitrogen), yielding the pSFV-E1 and pSFV-E2 constructs. Recombinant Semliki virus RNA synthesis, cell lysate preparation and immunoassays were then conducted as previously described [25]. For each serum sample tested, the optical density (OD) value obtained for wells with capture by ß-galactosidase protein was subtracted from that obtained for wells with capture by E1 or E2 proteins. The final data are expressed as the difference in OD (E1- or E2-ß-gal).

### Neutralization assays with HCV in cell culture

HCV in cell culture (HCVcc) harboring HCV envelope glycoproteins derived from genotype 1a (JFH1/H77), 1b (JFH1/J4), 2a (JFH1 WT), 3 (JFH1/S52) and 4 (ED43/JFH1) isolates [31–34], were used to assess and compare the neutralizing potential of the anti-HCV envelope protein antibodies present in pre- and post-vaccination rabbit sera, but also in two pairs of mixtures of equal quantities of an anti-E1 rabbit serum and an anti-E2 rabbit serum, and in these same individual sera. To prepare these mixtures, we selected from our previous immunization experiments [25] two animals from each group with sera presenting optimal cross-neutralizing properties against heterologous HCV strains of various genotypes. As the HCV genotype 4 is highly prevalent in some parts of the world [35] and there is a need for a prophylactic vaccine protecting against this particular genotype, we introduced the analysis of genotype 4 HCVcc neutralization into the last set of experiments, in addition to neutralization of the other genotypes, 1a, 1b, 2a, and 3. Infection levels were determined using a focus-forming unit (FFU) staining assay, as previously described [25]. The assay was performed in duplicate and the results are expressed as mean values.

### Statistical analyses

Statistical analyses were performed with nonparametric Mann-Whitney tests to evaluate the observed subtle differences in the immunogenetic potential and the capacity to induce neutralizing antibodies between immunization with a mixture of chimeric particles harboring E1 or E2 proteins separately and other vaccination strategies. A *p* value < 0.05 was considered to be statistically significant.

## Results

### Immunogenicity of the HCV E1 and E2 envelope glycoproteins presented separately or in the form of heterodimers at the surface of the chimeric HBV-HCV envelope particles

**Fig 1:**
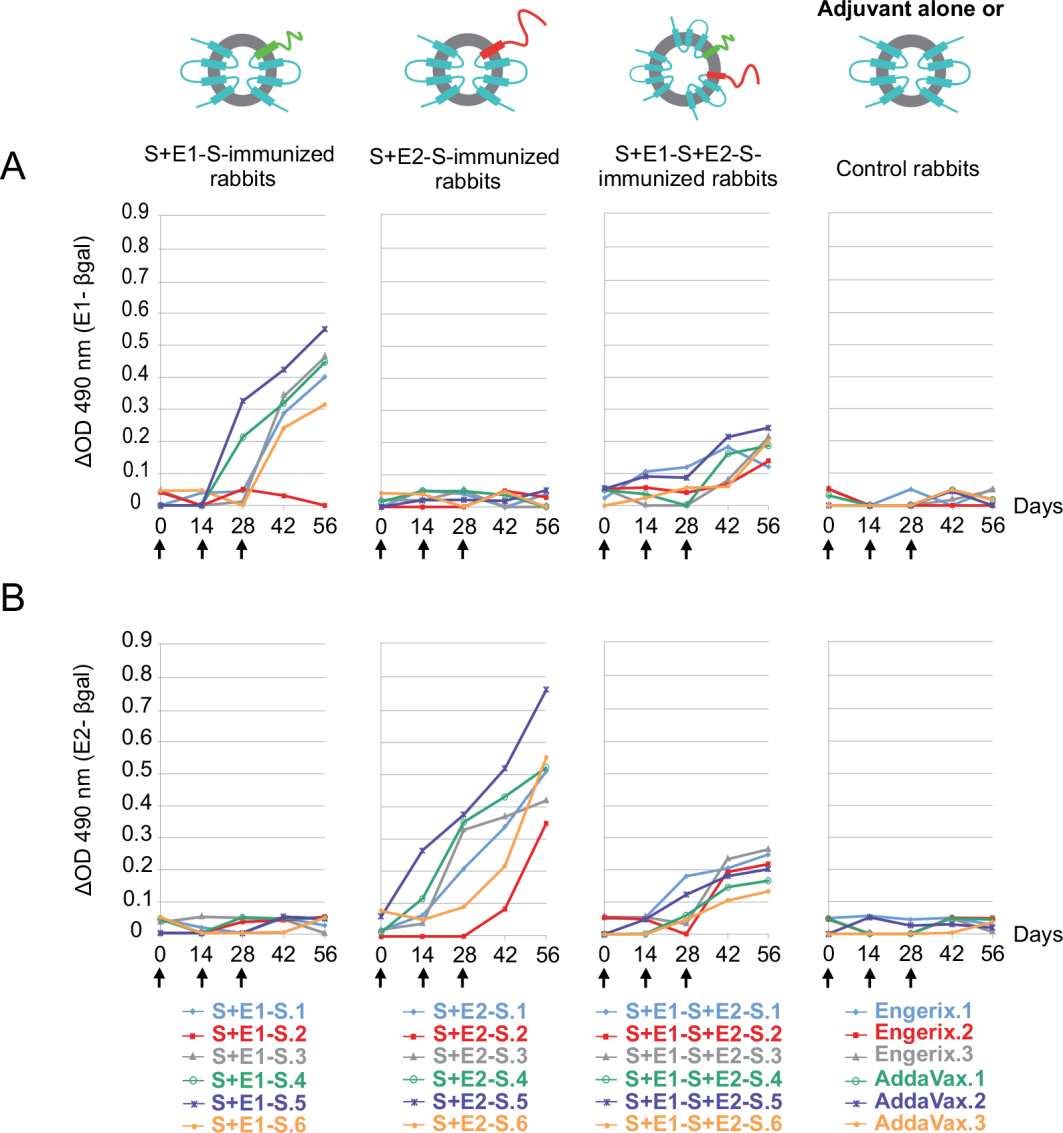
Detailed analysis of the antibody responses against the HCV E1 and E2 envelope glycoproteins induced in rabbits immunized with chimeric S+E1-S, S+E2-S or S+E1-S+E2-S particles. Specific anti-E1 (A) and anti-E2 (B) responses were evaluated with “in-house” ELISAs on rabbit sera collected at various time points [25]. Sera from rabbits immunized with the adjuvant alone or with the HBV vaccine Engerix were used as negative controls. Black arrows indicate the time at which immunization with 3 x 15 μg of immunogens occurred. Results are expressed as the difference in optical density (E1- or E2-β-gal).

We first evaluated, with biological material collected during a previous study [25], the separate contributions of the E1 and E2 envelope glycoproteins to the humoral immune responses induced in rabbits immunized with chimeric particles bearing E1, E2 or the heterodimer E1E2. These assays confirmed that chimeric particles harboring either the E1 or the E2 protein, separately, elicited high titers of anti-E1 or anti-E2 antibodies, but that the response induced by immunization with particles presenting E1 protein was much weaker than that induced by immunization with particles presenting E2 protein (Fig 1). Moreover, they clearly demonstrated that the individual anti-E1 and anti-E2 responses induced with particles presenting the E1E2 heterodimer were much weaker than those induced with chimeric particles presenting only E1 or E2. These results suggest that the immunogenicity of the two HCV envelope glycoproteins presented at the surface of the chimeric particles as a heterodimer was much weaker than that of the two envelope proteins exposed individually.

### Additive neutralizing properties of the anti-E1 and anti-E2 antibodies induced by immunization with chimeric HBV-HCV envelope particles

**Fig 2:**
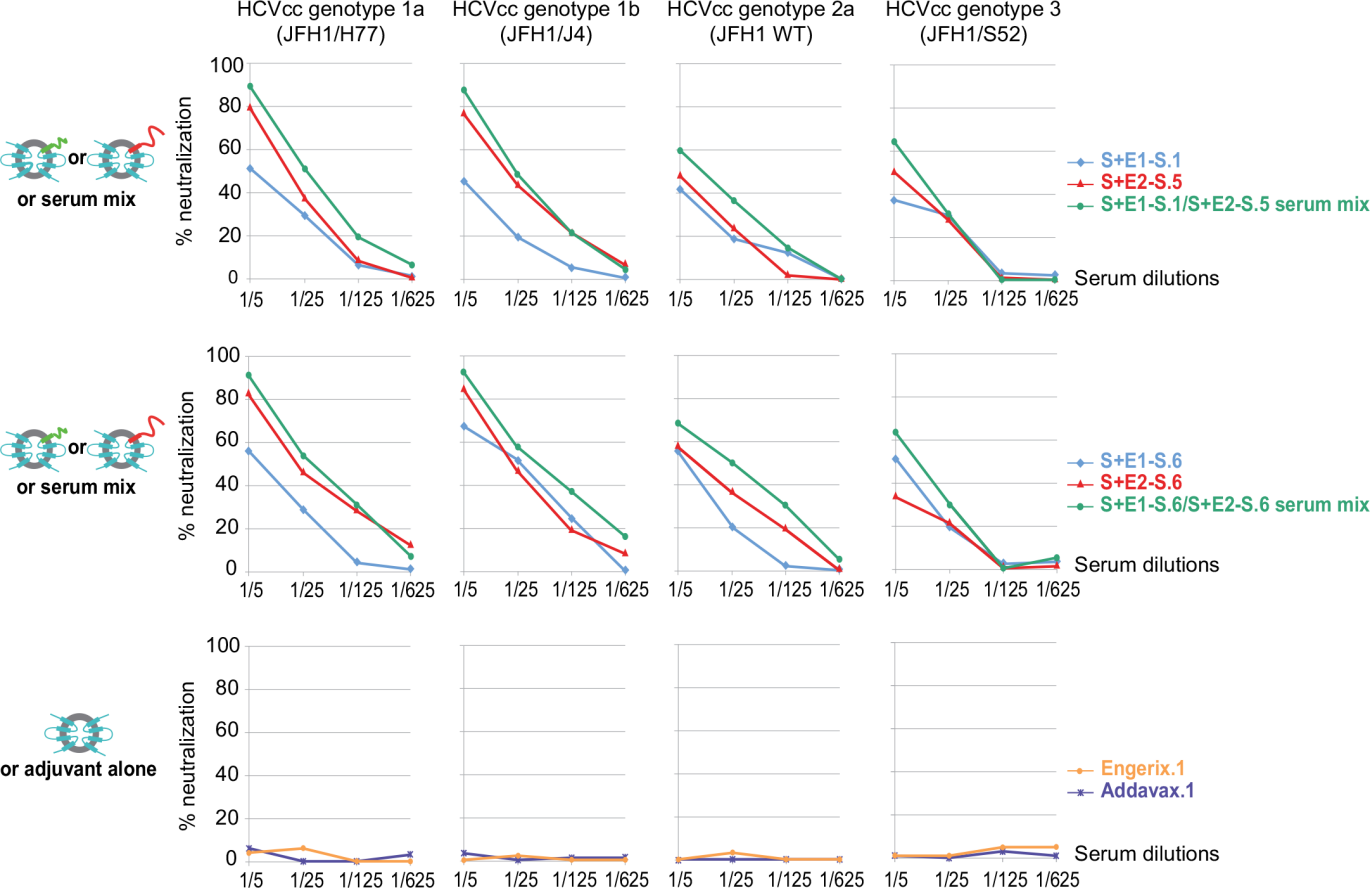
Comparison of the cross-neutralizing properties against HCVcc of two pairs of equal-amount mixtures of an anti-E1 rabbit serum and an anti-E2 rabbit serum with those of these sera used individually. HCVcc harboring HCV envelope glycoproteins derived from strains of various genotypes were first incubated for 1 hour at 37°C with five-fold serial serum dilutions of rabbit sera collected on day 56, or mixtures. To prepare these mixtures, we selected from our previous immunization experiments [25] two animals from each group with sera presenting optimal cross-neutralizing properties against heterologous HCV strains of various genotypes. Sera from rabbits immunized with the adjuvant alone or with Engerix were used as negative controls. They were then incubated with Huh7.5 cells for 6 hours. Infection levels were determined after 48 hours of incubation at 37°C, in a FFU staining assay. The percentage neutralization was determined by subtracting the infectious titer obtained with the pre-immune serum (day 0) from that obtained with the post-immune serum (day 56) from the same rabbit. The assay was performed in duplicate, and the results are expressed as mean values.

Our ultimate goal is to induce the best neutralizing response. We therefore investigated whether the anti-E1 and anti-E2 antibodies induced by immunization with our chimeric particles could have additive properties to increase the cross-neutralization of heterologous strains of various HCV genotypes. We compared the neutralizing properties of mixtures of equal quantities of an anti-E1 rabbit serum and an anti-E2 rabbit serum with those of these same individual sera against HCVcc harboring HCV envelope glycoproteins derived from genotype 1a, 1b, 2a and 3 isolates. In all cases, these mixed sera presented better cross-neutralizing properties in vitro against HCVcc harboring heterologous HCV envelope proteins derived from strains of different genotypes than did the individual sera (Fig 2). This cross-neutralizing potential was, nonetheless, less efficient for heterologous genotypes 2a and 3, which are genetically more distant. By contrast, sera from rabbits immunized with the HBV vaccine (Engerix) or adjuvant alone had no effect on the infectivity of HCVcc. These results highlight the additive neutralizing properties of the anti-E1 and anti-E2 antibodies, in HCV neutralization, and the importance of including both the E1 and E2 proteins in effective vaccination strategies, but in a configuration other than the E1E2 heterodimer.

### Strategy to induce an optimal dual anti-E1 and anti-E2 antibody response

**Fig 3:**
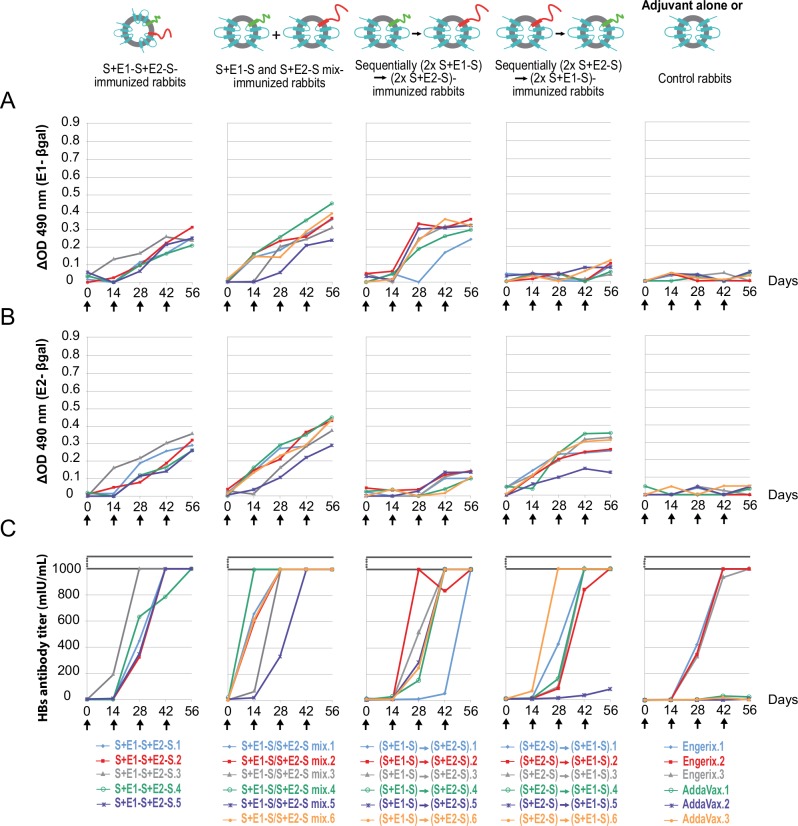
Humoral immune responses induced in rabbits immunized with chimeric particles harboring the E1E2 heterodimer, compared with a mixture or different sequential immunization combinations of chimeric particles harboring the E1 and E2 proteins separately. Specific anti-E1 (A) and anti-E2 (B) responses were evaluated with “in-house” ELISAs on rabbit sera collected at various time points. Anti-HBsAg responses (C) on these serum samples were evaluated with a routine immunoassay (Abbott Laboratories). Sera from rabbits immunized with the adjuvant alone or with Engerix were used as negative and positive controls, respectively. Black arrows indicate the time at which immunization with 4 x 15 μg of immunogens occurred. Results are expressed as the difference in optical density (E1- or E2-β-gal) and anti-HBsAg titer (mIU/ml), respectively.

Based on these findings for biological material collected during our previous animal immunizations, we conducted new immunization protocols with equal-amount mixtures or various sequential combinations of AddaVax-adjuvanted chimeric particles harboring E1 and E2 proteins separately, to try to identify the vaccination protocol inducing the best dual anti-E1 and anti-E2 response. Unfortunately, one of the rabbits immunized with particles bearing the E1E2 heterodimer died during blood sampling on day 14, but this death was clearly not related to the quality of the immunogen administered. All rabbits immunized by four injections of a mixture of chimeric particles bearing E1 and E2 separately were shown to develop sustained responses against both the E1 and E2 envelope proteins of HCV, whereas rabbits immunized with different sequential combinations (two immunizations with particles bearing only E1 followed by two immunizations with particles bearing only E2, and vice versa) essentially developed a response against the first immunogen (Fig 3A and 3B). The mixture of chimeric particles harboring E1 and E2 proteins separately induced stronger specific antibody responses against both E1 and E2 than the chimeric particles presenting the E1E2 heterodimer. All rabbits presenting humoral anti-E1 and anti-E2 responses also displayed a strong humoral anti-HBsAg response, equivalent to that observed in rabbits immunized with the commercial HBV vaccine Engerix (Fig 3C).

### Analysis of the additive neutralizing properties of antibodies induced by immunization with the mixture of HBV-HCV chimeric envelope particles harboring E1 and E2 proteins separately

**Fig 4:**
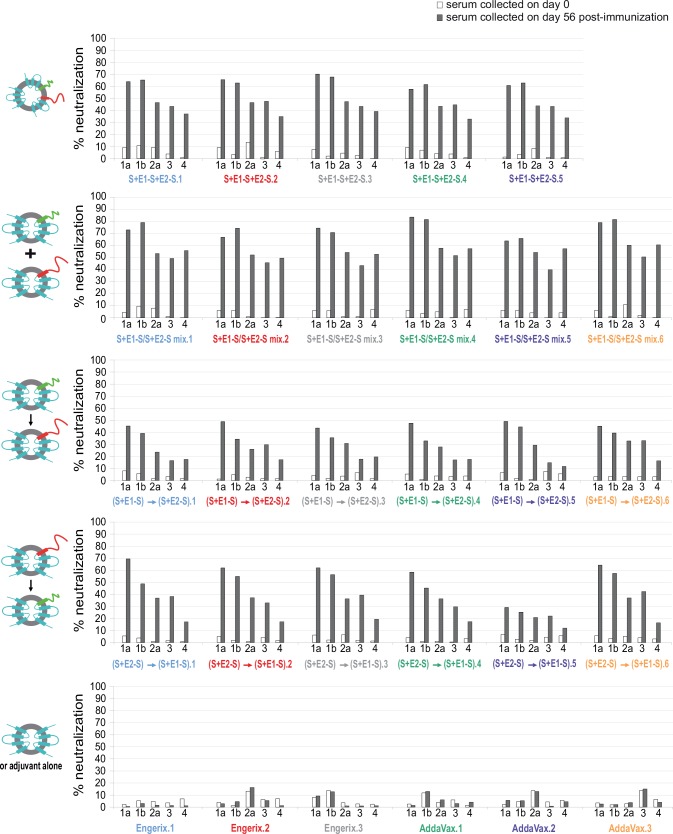
Cross-neutralizing properties against HCVcc of antibodies induced by immunization with chimeric particles harboring the E1E2 heterodimer, compared with those of antibodies induced by a mixture or different sequential immunization combinations of chimeric particles harboring the E1 and E2 proteins separately. Five-fold dilutions of rabbit sera collected on days 0 and 56 were first incubated with HCVcc harboring HCV envelope glycoproteins derived from strains of various genotypes for 1 hour at 37°C, which were then used to infect Huh7.5 cells for 6 hours. Infection levels were determined after 48 hours of incubation at 37°C, in a FFU staining assay. The percentage neutralization in the presence of the post-immune serum (day 56) was compared with that in the presence of pre-immune serum (day 0) from the same rabbit. The assay was performed in duplicate and the results are expressed as mean values.

After evaluating the humoral immune responses of vaccinated animals, we further tested the neutralizing properties of their sera against infections with HCVcc derived from different heterologous HCV genotypes. These analyses demonstrated that vaccination strategy in which anti-E1 and anti-E2 antibodies were induced by immunization with a mixture of particles harboring the E1 and E2 proteins separately had the better cross-neutralizing properties in vitro against HCVcc harboring heterologous HCV envelope proteins derived from strains of genotypes 1a, 1b, 2a, 3 and 4 (Fig 4). As expected from the analysis of the anti-E1 and anti-E2 humoral response, the neutralization obtained with sera from animals vaccinated by sequential immunizations with the two HCV envelope proteins gave disapointing results. Interestingly, the gain in the percentage of HCVcc neutralization obtained by vaccination with the mixture of particles was significant with respect to the other vaccination strategies and genotypes in all but one case (Table 1). Indeed, other than for genotype 3, this gain over particles bearing the E1E2 heterodimer was highly significant, for genotypes 2a and 4 (*p* < 0.01), and significant, for genotypes 1a and 1b (*p* < 0.05). Moreover, additional neutralizing assays using five-fold serial dilutions of rabbit sera collected on days 0 and 56 demonstrated that a significant specific neutralizing activity against HCVcc could still be observed for high serum dilutions (S3 Fig).

**Table 1:** Comparison of the different vaccination strategies in terms of their capacity to induce neutralizing antibodies against various HCVcc genotypes.

	HCVcc genotype neutralization (% median)				
	1a	1b	2a	3	4
**Mix of S+E1-S & S+E2-S (particles bearing E1 and E2 separately)**	68.75	70.16	49.40	46.71	52.39
**S+E1-S+E2-S (particles with E1E2 heterodimers)**	56.25 *	59.26 *	37.30 **	40.85 (NS)	32.98 **
**(S+E1-S then S+E2-S sequential immunization)**	42.12 **	34.39 **	25.14 **	12.50 **	14.96 **
**(S+E2-S then S+E1-S) sequential immunization**	56.85 *	48.55 **	34.10 **	32.95 **	14.57 **
**Engerix or AddaVax alone**	0 **	0.29 **	0.58 **	0 **	0 **

Nonparametric Mann-Whitney tests (statistical analysis performed by comparing the median percentage neutralization obtained by immunization with the mixture of particles bearing E1 and E2 separately, with that obtained by other vaccination strategies)

(NS) non-significant

(*) p<0.05

(**) p<0.01

## Discussion

The HCV envelope glycoproteins E1 and E2, presented in the correct conformation on the surface of viral particles, are clearly targets for neutralizing antibodies [36–39] and are thus attractive immunogens for the development of a prophylactic vaccine for HCV [40], but their ideal model of processing and presentation for effective vaccination must be determined. Moreover, the importance of the E1 protein in the development of such a vaccine remains a matter of debate. Some studies have reported E1 to be only weakly immunogenic or to induce non-neutralizing antibodies [41, 42]. However, there are several lines of evidence to suggest that this envelope protein is a particularly interesting target for neutralizing antibody induction: (i) E1 displays a higher degree of intergenotype cross-reactivity than E2, which varies considerably between strains, especially in its hypervariable regions [22]; (ii) anti-E1 monoclonal cross-neutralizing antibodies have already been reported [36, 38]; (iii) antibody responses to the E1 envelope protein are largely impaired in patients chronically infected with HCV [7, 43], suggesting that such responses might facilitate effective vaccination and HCV clearance; (iv) anti-E1 antibodies induced by immunization with a recombinant form of E1 have been shown to confer protection against experimental infection with heterologous HCV strains in chimpanzees [44]. Retroviral particles pseudotyped for HCV E2 and/or E1 envelope proteins used in a prime-boost strategy with HCV-recombinant measles virus and adenovirus-based recombinant vectors for priming, have recently been shown to induce high titers of anti-E2 and/or anti-E1 antibodies, and of cross-neutralizing antibodies, in both mice and macaques [42]. Interestingly, this study revealed that an anti-E1 response is not only difficult to trigger, consistent with the observation that anti-E1 antibodies are rarely detected in significant quantities in patients [7, 43], but also that it requires the dissociation of E1 and E2 expression. This suggests that E2 is immunodominant or that immunogenic E1 epitopes are masked in the presence of E2.

With the aim of developing an affordable and easy-to-produce HCV vaccine, we recently developed an original strategy for incorporating entire wild-type genotype 1a HCV E1 and/or E2 proteins into HBV envelope subviral particles [24]. We previously reported that chimeric HBV-HCV subviral envelope particles harboring either a genotype 1a E1 protein or a genotype 1a E2 protein elicited strong specific antibody responses to the HCV and HBV envelope proteins in immunized animals, including high titers of cross-neutralizing anti-E1 or anti-E2 antibodies, highlighting the importance of including both envelope proteins for an effective vaccination strategy [25]. These neutralizing antibodies inhibited E2/CD81 binding only partially, suggesting action at both pre- and post-binding entry steps. Unexpectedly, although both E1 and E2 proteins exposed individually at the surface of the chimeric particles elicited cross-neutralizing antibodies, no cooperation between these proteins was obtained when they were presented together in the form of the E1E2 heterodimer at the surface of the chimeric particles.

We used this HBV-HCV chimeric envelope particle model in this study, in which we demonstrated a profound impairment of the specific anti-E1 and anti-E2 antibody responses induced in rabbits immunized with particles harboring the E1 and E2 proteins in the form of a heterodimer. Much strong antibody responses were obtained in rabbits immunized with particles harboring E1 and E2 separately, and particularly in rabbits immunized with an equal-amount mixture of these particles. In contrast, all rabbits immunized with different sequential combinations failed to develop sustained responses against both the E1 and E2 envelope proteins of HCV, as if we were witnessing a profound disturbance of the immune system, which does not seem to recognize and take into consideration, or very tardily, the second highly different immunogen. These observations confirm that these proteins are less immunogenic when presented in this heterodimer conformation, confirming data from previous studies suggesting that it is difficult to induce an anti-E1 response, with significant levels of anti-E1 antibodies obtained only if E1 is dissociated from E2 [22, 42]. This phenomenon may be explained by the masking of certain immunodominant epitopes by the productive folding of E1 and E2 into a heterodimer at the surface of the chimeric particles. To confirm this hypothesis, it will be interesting in future investigations to determine the epitopes recognized by the neutralizing antibodies produced in response to immunization with our vaccine particles. Moreover, such information could allow the identification of the minimum E1 and E2 epitopes having cross-neutralizing properties. This study is the first to analyze and compare the immunogenicity and neutralizing antibody-inducing properties of the entire E1 and E2 proteins and the E1E2 heterodimer. Several studies have tried to determine whether antibodies induced by E1, E2 or the E1E2 heterodimer can mediate HCV neutralization. However, all these studies were conducted with modified envelope proteins, making comparisons with entire wild-type HCV E1 and E2 proteins difficult. Indeed, several studies were conducted with envelope proteins (E1 or E2 or E1E2 heterodimer) deleted of their transmembrane domain [20–23, 45, 46] or their hypervariable regions [44], to reveal novel structural features and to create new targets for the induction of broad neutralizing antibodies. In other studies, ectodomains of E1 and/or E2 were fused to the TMD and the cytoplasmic region of the measles virus fusion protein [47] or the vesicular stomatitis virus G protein [42]. In addition, the immunogenicity of the separate envelope proteins has not systematically been compared with that of the E1E2 heterodimer, making it difficult to draw definitive conclusions. We also show, for the first time, that the anti-E1 and anti-E2 antibodies induced by immunization with a mixture of chimeric particles harboring HCV E1 and E2 separately had additive neutralizing properties that increase the cross-neutralization of heterologous strains of various HCV genotypes. Although this gain in the percentage of HCVcc neutralization does not appear very important, it is nonetheless significant and needs to be taken in consideration for optimization of HCV envelope-based vaccine strategies. Moreover, this finding highlights the importance of including both E1 and E2, in a configuration other than the E1E2 heterodimer, for an effective vaccination strategy. This may be because such strategies result in higher titers of anti-E1 and anti-E2 antibodies. However, it may also reflect the low proportion of E1E2 heterodimers expressed at the surface of HCV virions [48], where HCV E1 and E2 envelope proteins tend to form large covalent complexes stabilized by disulfide bridges. It is possible that the antibodies induced by E1 and E2 as separate immunogens are more able to interact with these complexes and then act as neutralizing antibodies than those induced by the E1E2 heterodimer. However, further investigations are required to confirm this hypothesis.

In conclusion, our findings have important implications for the optimization of HCV vaccination strategies based on HCV envelope proteins, regardless of the platform used to present these proteins to the immune system. In our HBV-HCV chimeric envelope particles, the HCV E1 and E2 envelope proteins need to be presented separately (i.e. not as a heterodimer), and used as a mixture to induce an optimal dual anti-E1 and anti-E2 response neutralizing heterologous strains of various HCV genotypes. Further studies, including immunization in humans, will be required before definitive conclusions can be drawn. However, these vaccine particles, which could be produced by industrial procedures adapted from those established for the HBV vaccine, appear promising for use in an affordable potential prophylactic vaccine against HCV and, potentially, a bivalent HBV-HCV vaccine.

## Supporting Information

S1 Fig Analysis of the final chimeric HBV-HCV envelope particle solutions used as immunogens. (A), Western blots probed with polyclonal anti-HBsAg (R247) and monoclonal anti-E1 (A4) and anti-E2 (H52) antibodies demonstrated the efficient incorporation of chimeric HBV-HCV envelope proteins (E1-S or/and E2-S) into the secreted S+E1-S, S+E2-S and S+E1-S+E2-S subviral particles used for the preparation of the final immunogen solutions. (B), An immunoassay using human monoclonal antibodies recognizing conformation-dependent discontinuous epitopes on the E2 protein (AR3A) and the folded E1-E2 complex (AR5A) demonstrated the productive folding and heterodimerization of HCV envelope proteins in the context of fusion proteins. The monoclonal antibodies AR3A and AR5A were added to ELISA wells coated with chimeric particles (S, S+E1-S, S+E2-S, S+E1-S+E2-S or a S+E1-S/S+E2-S mixture; 25 μg/ml) or JFH-1 WT viruses purified on sucrose cushions (1,000 focus-forming units; positive control: gray histogram) captured with a lectin precoating. Specific binding was detected with monoclonal peroxidase-conjugated mouse anti-human immunoglobulin antibody. Absorbance at 490 nm was determined. The data shown are the means ± standard deviation of one experiment performed in quadruplicate.
(EPS)

S2 Fig Immunization protocol design. Groups of six rabbits were immunized subcutaneously four times with AddaVax-adjuvanted chimeric S+E1-S+E2-S envelope particles (vaccine doses consisting of 15 micrograms of the S+E1-S+E2-S immunogen), or with a mixture (vaccine doses consisting of equal amounts (7.5 micrograms) of the S+E1-S and S+E2-S subviral particles produced separately) or different sequential immunization combinations of AddaVax-adjuvanted chimeric particles harboring E1 or E2 proteins separately (vaccine doses consisting of 15 micrograms of the S+E1-S or S+E2-S immunogens), on days 0, 14, 28 and 42. For sequential immunization combinations, rabbits were either immunized twice with S+E1-S particles and then twice with S+E2-S particles, or vice versa. Groups of three rabbits immunized with the Addavax adjuvant alone or with Engerix (vaccine doses consisting of 15 micrograms of the S immunogen), a commercially available HBV vaccine, were used as negative and positive controls, respectively. Serum samples were collected from rabbits at various time points (days 0, 14, 28, 42, and 56), to characterize the antibody responses.
(EPS)

S3 Fig Cross-neutralizing properties against HCVcc of five-fold serial serum dilutions of rabbits immunized either with chimeric particles harboring the E1E2 heterodimer or a mixture or different sequential immunization combinations of chimeric particles harboring the E1 and E2 proteins separately. Dilutions of rabbit sera collected on days 0 and 56 were first incubated with HCVcc harboring HCV envelope glycoproteins derived from strains of various genotypes for 1 hour at 37°C, which were then allowed to infect Huh7.5 cells for 6 hours. After 48 hours of incubation at 37°C, the percentage neutralization was determined by subtracting the infectivity titer obtained with pre-immune serum (day 0) from that obtained with post-immune serum (day 56). The assay was performed in duplicate, and the results are expressed as mean values.
(EPS)
